# Wearable Augmented Reality for Nystagmus Examination in Patients With Vertigo: Randomized Crossover Usability Study

**DOI:** 10.2196/75327

**Published:** 2025-11-11

**Authors:** Ching-Nung Wu, Ming-Che Chen, Chien-Yan Chien, Hsiang-Han Chang, Sheng-Dean Luo, Chung-Feng Hwang, Wan-Jung Chang

**Affiliations:** 1 Department of Otolaryngology Kaohsiung Chang Gung Memorial Hospital Kaohsiung Taiwan; 2 School of Traditional Chinese Medicine College of Medicine Chang Gung University Taoyuan Taiwan; 3 Department of Otolaryngology Kaohsiung Municipal Ta-Tung Hospital Kaohsiung Taiwan; 4 Department of Electronic Engineering Southern Taiwan University of Science and Technology Tainan Taiwan; 5 Graduate Institute of Clinical Medical Sciences College of Medicine Chang Gung University Taoyuan Taiwan; 6 School of Medicine College of Medicine National Sun Yat-sen University Kaohsiung Taiwan; 7 Department of Electronic Engineering National Kaohsiung University of Science and Technology kaohsiung Taiwan

**Keywords:** nystagmus examination, augmented reality, oculomotor test, video-oculography, vertigo diagnostics

## Abstract

We demonstrate the feasibility of a wearable, augmented reality–based nystagmus examination system, showing its preliminary diagnostic agreement with conventional video-oculography and its potential for portable vestibular assessment in patients with vertigo.

## Introduction

Vertigo commonly arises from benign vestibular dysfunction but may also have a central cause such as stroke (in approximately 10% of cases) [1]. Nystagmus analysis is key to differentiating these disorders [2], yet conventional video-oculography (VOG) requires specialized laboratories and personnel, limiting access [3]. We developed a wearable augmented reality (AR)–based system delivering standardized oculomotor stimuli with real-time eye tracking. This study reports its design and preliminary validation.

## Methods

### Overview

This feasibility study evaluated the usability, accuracy, and tolerability of a wearable AR-based nystagmus system in a hospital clinical setting. The system integrated hardware and software to simulate conventional oculomotor testing with real-time eye tracking and automated data processing.

For comparison, the VNG Ulmer system (Synapsys; Multimedia Appendix 1) performs 6 standardized tests of 3 stimulus types: (1) gaze-evoked nystagmus at plus or minus 15° (60 s total; horizontal/vertical axes), (2) saccades with fixed displacements every 4 seconds over 30 seconds (8-9 trials; horizontal/vertical axes), and (3) smooth pursuit at 0.25 Hz for 30 seconds (7-8 cycles; horizontal/vertical axes) [4].

The wearable AR system (Multimedia Appendix 2) comprised J7EF Gaze smart glasses, an Android-based portable device (APD), and a back-end platform. The structural components included dual Si-OLED displays, a 30 Hz infrared eye-tracking sensor, and an optional magnetic light shield to replicate Frenzel goggles (Multimedia Appendix 3). The APD, connected via USB Type-C, ran Unity 3D software to generate a virtual 1-meter display.

**Figure 1:**
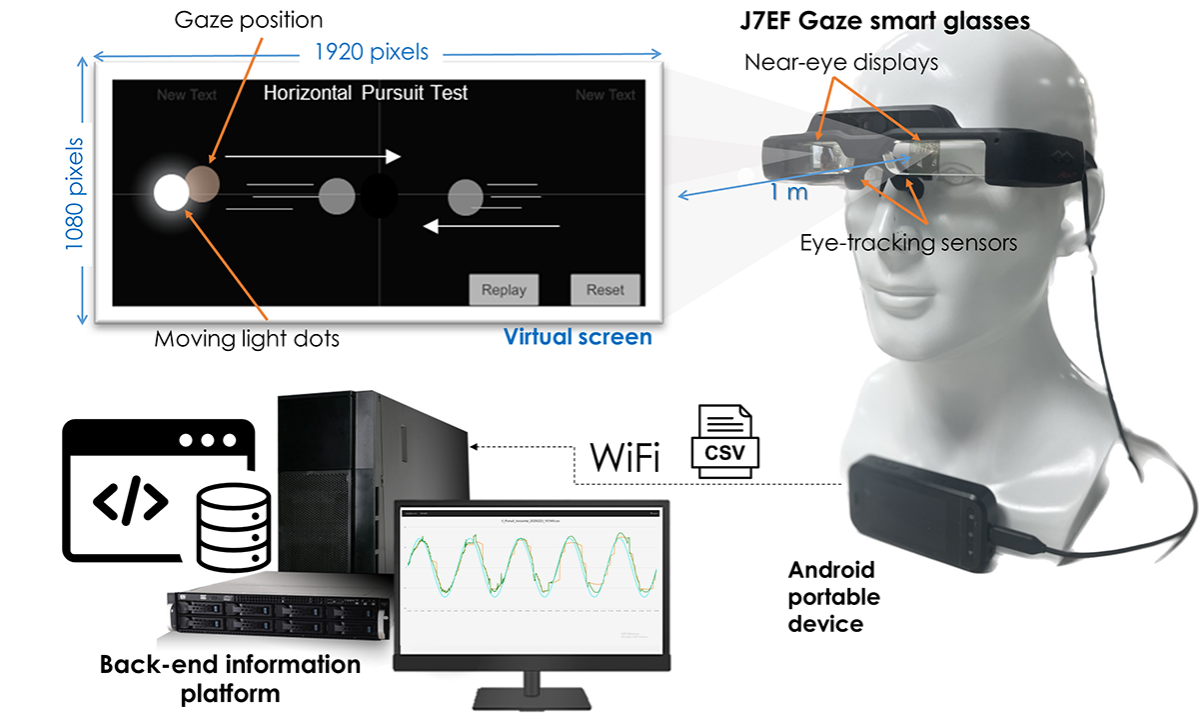
This figure illustrates the architecture of the wearable augmented reality–based nystagmus examination system. The J7EF Gaze smart glasses incorporate near-eye displays and infrared eye-tracking sensors to present standardized visual stimuli and capture real-time gaze positions. A virtual screen simulates a 1-meter viewing distance, displaying moving light dots for oculomotor assessment. Data are processed on an Android portable device and transmitted via Wi-Fi to a back-end information platform for visualization and further analysis.

In-house software delivered 6 standardized stimuli (fixation, saccades, and smooth pursuit in the horizontal and vertical axes), consistent with the VOG protocol. Real-time gaze data were transmitted via Wi-Fi for secure storage and automated analysis. This setup reproduced conventional vestibular assessments while enabling portable, automated nystagmus evaluation (Figure 1).

### Participants and Study Procedures

**Figure 2:**
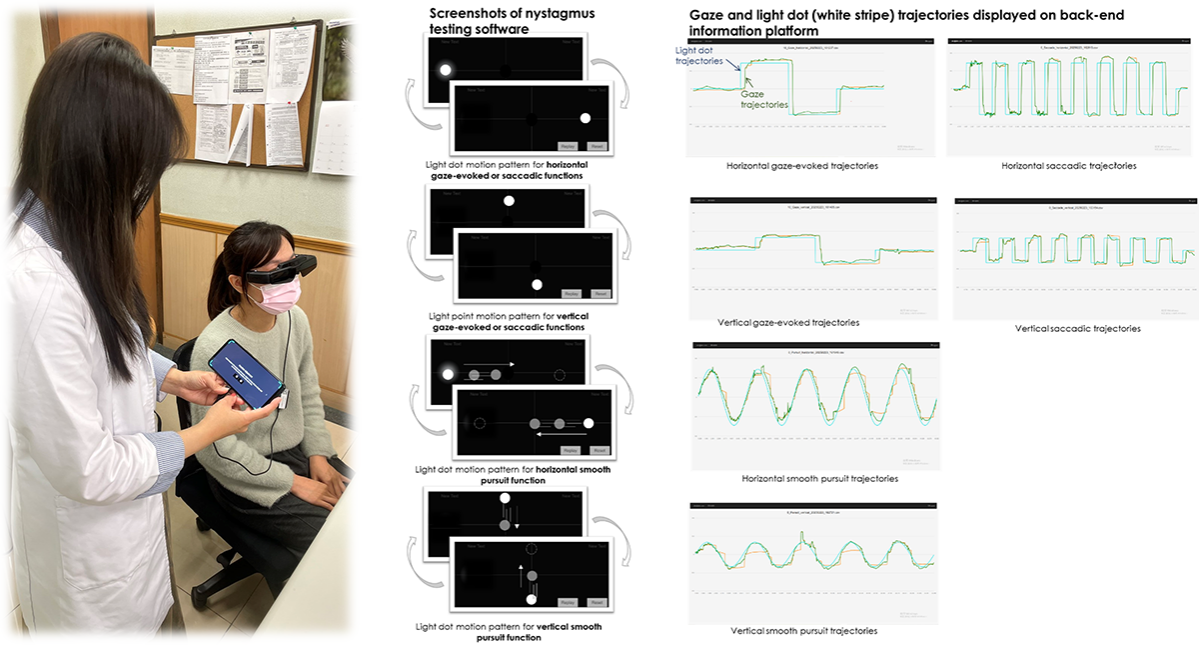
This figure illustrates the workflow of the augmented reality–based oculomotor examination, including software-generated visual stimuli and real-time eye movement tracking. The light dot, white stripe, and gaze trajectories are displayed on the back-end information platform, enabling comparative analysis of expected vs actual eye movement responses.

Nine patients with vertigo were enrolled (October 2024 to January 2025); 8 completed both AR and VOG examinations in a randomized crossover design with a 30-minute washout (Figure 2). After each examination, participants rated discomfort using a visual analog scale (VAS; range 0-10). All waveform outputs were pooled and blindly interpreted by a board-certified otologist. The primary outcome was diagnostic concordance, quantified as percentage agreement [5]. Secondary outcomes were VAS scores and diagnostic performance metrics (accuracy, sensitivity, specificity, positive predictive value [PPV], and negative predictive value [NPV]), each reported with 95% CIs (Clopper-Pearson exact method).

### Ethical Considerations

This study was approved by the institutional review board of Kaohsiung Chang Gung Memorial Hospital (202202194B0C501). All participants provided written informed consent. Data were anonymized, encrypted, and securely stored. No compensation was given.

## Results

One participant with prior cataract surgery could not calibrate the AR glasses, leaving 8 valid cases (mean age 60.4, SD 8.9 years; range 46-72). No significant discomfort or adverse effects were reported (Multimedia Appendix 4).

A total of 48 oculomotor data points were analyzed (saccades, pursuit, and gaze fixation). Agreement rates between AR and VOG ranged from 62.5% to 87.5% (Multimedia Appendix 5). Overall diagnostic accuracy was 77.1%, with sensitivity 81.8% (9/11; 95% CI 48.2%-97.7%), specificity 75.7% (28/37; 95% CI 58.8%-88.2%), PPV 50.0% (9/18; 95% CI 26.0-73.9%), and NPV 93.3% (28/30; 95% CI 77.9%-99.2%) (Multimedia Appendix 6). For central pathology, sensitivity reached 83.3% and specificity 100%. VAS scores did not differ significantly between AR and VOG, confirming tolerability.

## Discussion

This study demonstrates the feasibility of wearable AR glasses for nystagmus examination, showing diagnostic consistency comparable to VOG, particularly in ruling out central abnormalities. Results align with recent AR-based HINTS (head impulse, nystagmus, test of skew) assessments using head-mounted devices [6] and smartphone nystagmus apps with approximately 82% sensitivity [7].

Although enrollment spanned 4 months, only 8 participants completed paired assessments due to the 2-to-3-month delay for conventional VOG. This explains the small sample and illustrates the clinical bottleneck the AR system seeks to address. Patient tolerance was favorable, with no significant discomfort, consistent with prior AR studies [8]. One participant with cataract surgery could not be calibrated, likely due to altered ocular optics, underscoring the need for adaptive algorithms [9]. Unlike stationary VOG laboratories, wearable AR systems are portable and deployable in clinics, emergency care, or telemedicine, enabling point-of-care testing without specialized infrastructure [10]. With automated guidance, real-time tracking, and potential artificial intelligence integration, they may reduce reliance on experts and support decision-making.

Limitations include the small sample, yielding wide CIs for sensitivity (81.8%; 48.2%-97.7%) and specificity (75.7%; 58.8%-88.2%), reducing precision. The moderate PPV (50.0%; 26.0%-73.9%) highlights the need for improved processing. Diagnostic concordance was measured by percent agreement, which does not adjust for chance; future studies should apply Cohen κ and multiple raters. Finally, as a single-center pilot with one clinician, multicenter validation is required to confirm generalizability.

## Appendix

Multimedia Appendix 1 Full conventional video-oculography protocol, including test parameters and classification criteria.

Multimedia Appendix 2 Detailed system design and hardware/software specifications.

Multimedia Appendix 3 Structural components of the J7EF Gaze smart glasses.

Multimedia Appendix 4 Patient characteristics and oculomotor examination results.

Multimedia Appendix 5 Heatmap of agreement rates between augmented reality and video-oculography signals.

Multimedia Appendix 6 Confusion matrix of augmented reality–based vs video-oculography–based classifications of suspected central vestibular pathology.

## Abbreviations

APD: Android-based portable device
AR: augmented reality
HINTS: head impulse, nystagmus, test of skew
NPV: negative predictive value
PPV: positive predictive value
VAS: visual analog scale
VOG: video-oculography

## Data Availability

All deidentified data generated or analyzed during this study are included in this published article and its supplementary files.
