# Supplementary material for: Wearable Augmented Reality for Nystagmus Examination in Patients With Vertigo: Randomized Crossover Usability Study
Source: J Med Internet Res. 2025 Nov 11;27:e75327. doi: 10.2196/75327 (PMC12648123; doi:10.2196/75327)
Supplement: Multimedia Appendix 1 [file jmir_v27i1e75327_app1.docx]

**Multimedia Appendix 1. Full Conventional VOG Protocol, including test parameters and classification criteria**

A VOG test battery was performed using VOG Ulmer system (Synapsys, Mountain View, CA, USA); this includes a number of tests, including of spontaneous nystagmus with or without fixation, gaze-evoked nystagmus, positional and positioning nystagmus, saccades, smooth pursuit, and optokinetic nystagmus, as well as caloric testing. In this study, only the oculomotor tests, including six dimensions (gaze-evoked nystagmus (horizontal/vertical axes), saccades (horizontal/vertical axes), and smooth pursuit (horizontal/vertical axes) were measured for an individual subject.

The results of the oculomotor study were classified as either indicative of central vestibular disorder or nonspecific findings. Oculomotor signals within individual dimensions were labeled abnormal if they indicated central vestibular disorder and labeled normal if showing nonspecific findings. Central vestibular disorder was considered present on the basis of the results of the following tests, which the consensus of detailed criteria had been reached: [1]

• Gaze-evoked nystagmus (horizontal or vertical axis): The patient was made to look front, left, right, up, and down at angles of 15 for 20 s at one side, and the nystagmus was recorded. The whole interpretation range for one dimension was 60 s. Pure torsional or vertical nystagmus, direction-changing nystagmus, and gaze-evoked nystagmus opposite to Alexander’s law were considered as central vestibular findings.

• Saccades (horizontal or vertical axis): Targets were displaced in a predefined alternating pattern between ±15°, rather than randomly, ensuring fixed displacement across trials. Each displacement occurred at roughly 4‑second intervals over a 30‑second window, yielding 8–9 repetitions per dimension. Patients were instructed to follow the stimulus only with their eyes while keeping their head stable. Latency, defined as the delay between target onset and initiation of eye movement, was considered abnormal if consistently >260 ms. Precision referred to the amplitude of the eye movement relative to the target; hypometria of 10–20% was considered normal. Asymmetrical saccades indicated abnormality.

•Smooth pursuit (horizontal or vertical axis): Patients tracked a bright spot moving sinusoidally across the horizontal or vertical plane at a frequency of 0.25 Hz. The trajectory was centered at 0° and oscillated between ±15° (i.e., a 15° amplitude from center). Each trial lasted 30 seconds, encompassing 7–8 complete cycles. Pursuit was classified as abnormal if the trajectory appeared saccadic or jerky. Symmetrically impaired pursuit was considered a nonspecific central finding, whereas asymmetrically impaired pursuit suggested a unilateral hemispheric or posterior fossa lesion.

[1] Mekki, S. The role of videonystagmography (VNG) in assessment of dizzy patient. Egypt. J. Otolaryngol. 2014, 30, 69–72.
