# Supplementary material for: Wearable Augmented Reality for Nystagmus Examination in Patients With Vertigo: Randomized Crossover Usability Study
Source: J Med Internet Res. 2025 Nov 11;27:e75327. doi: 10.2196/75327 (PMC12648123; doi:10.2196/75327)
Supplement: Multimedia Appendix 2 [file jmir_v27i1e75327_app2.docx]

**Multimedia Appendix 2. Detailed system design and hardware/software specifications**

The wearable AR system is composed of the J7EF Gaze smart glasses [1], an Android-based Portable Device (APD), and a back-end information platform, as shown in Figure 1. The J7EF Gaze smart glasses feature dual Si-OLED near-eye displays and a 30 Hz infrared eye-tracking sensor. A magnetic light-blocking shield can be attached to simulate dark testing conditions, similar to clinical oculomotor assessments with Frenzel goggles. The glasses are connected to the APD via USB Type-C. The APD runs custom nystagmus testing software developed using the Unity 3D framework [2].

**Display specifications and visual angles:**

The AR device projects a fixed virtual screen with a diagonal size of 120 inches at a distance of approximately 2.5 meters from the eye (default device setting). At this distance, the effective field of view covers about ±28° horizontally and ±17° vertically. To ensure symmetrical stimulus presentation across horizontal and vertical axes despite the difference in field of view, all oculomotor stimuli in this study were standardized to a ±15° range. This range fits within both the horizontal and vertical limits of the device and avoids distortion. No additional geometric correction was applied. Instead, all participants underwent a built-in eye-tracking calibration procedure before testing to align gaze position with the projected virtual screen, thereby ensuring that stimuli at ±15° were perceived correctly across individuals.

**Stimulus presentation:**

The testing software presents six specific oculomotor stimuli: horizontal and vertical gaze-evoked, saccadic, and smooth pursuit.

- Gaze-evoked: Light dots remained at ±15° horizontally or vertically for 20 seconds per direction.
- Saccades: Light dots shifted in a predefined alternating pattern between +15° and –15° every 4 seconds, repeated 8–9 times within a 30-second trial.
- Smooth pursuit: Light dots followed a sinusoidal trajectory centered at 0° and oscillating between ±15° at 0.25 Hz (7–8 cycles over 30 seconds).

**Data acquisition and storage:**

Real-time gaze positions from the eye-tracking sensor were recorded simultaneously with the stimulus trajectory. The APD, equipped with a WiFi module, transmitted both sets of data to the back-end platform for storage and automated analysis.

In summary, the AR system replicates conventional vestibular assessments by projecting standardized stimuli within a controlled ±15° field, combined with real-time gaze tracking, calibration, and data storage. This ensured consistency between intended and perceived stimulus angles, despite the asymmetrical horizontal and vertical field of view inherent to the hardware.

[1] JORJIN, “J-Reality J7EF Gaze,” 2022. [Online] Available: https://www.jorjin.com/products/ar-smart-glasses/j-reality/j7ef-gaze/

[2] Unity Technologies, “Unity – Real-time 3D development platform,” [Online]. Available: https://unity.com. [Accessed: Mar. 1, 2025].
