# Supplementary material for: Wearable Augmented Reality for Nystagmus Examination in Patients With Vertigo: Randomized Crossover Usability Study
Source: J Med Internet Res. 2025 Nov 11;27:e75327. doi: 10.2196/75327 (PMC12648123; doi:10.2196/75327)

**Multimedia Appendix 3. Structural components of the J7EF Gaze smart glasses (Jorjin Technologies Inc., Model J7EF, Taiwan), showing dual Si-OLED displays for visual stimuli presentation, a 30 Hz infrared eye-tracking sensor, and a magnetic light-blocking shield to simulate dark testing conditions.**


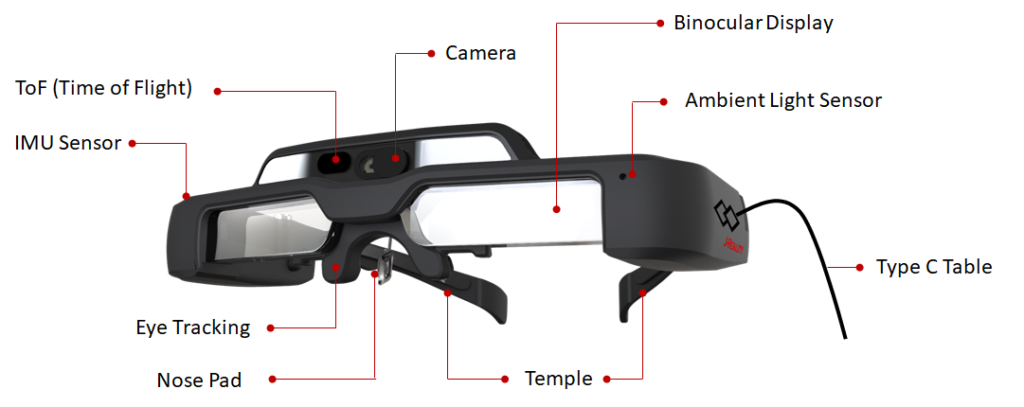

Supplement: Multimedia Appendix 3 [file jmir_v27i1e75327_app3.docx]
