# Supplementary material for: Wearable Augmented Reality for Nystagmus Examination in Patients With Vertigo: Randomized Crossover Usability Study
Source: J Med Internet Res. 2025 Nov 11;27:e75327. doi: 10.2196/75327 (PMC12648123; doi:10.2196/75327)
Supplement: Multimedia Appendix 4 [file jmir_v27i1e75327_app4.docx]

**Multimedia Appendix 4. Patient Characteristics and Oculomotor Examination Results**

| No. | Sex | Age | Symptoms | Duration | Sequence | Augmented reality (AR)-based testing | | | | | | | | Video-oculography (VOG) system | | | | | | | |
| --- | --- | --- | --- | --- | --- | --- | --- | --- | --- | --- | --- | --- | --- | --- | --- | --- | --- | --- | --- | --- | --- |
|  |  |  |  |  |  | Central | SacH | SacV | PurH | PurV | GazH | GazV | VAS | Central | SacH | SacV | PurH | PurV | GazH | GazV | VAS |
| 1 | F | 68 | vertigo | 5 hours | VOG | + | + | + | + | + | - | - | 0 | + | - | - | + | + | - | - | 0 |
| 2 | F | 70 | dizziness | 4 months | VOG | + | - | - | + | + | - | - | 0 | + | - | - | - | + | - | - | 0 |
| 3 | F | 72 | dizziness | 6 months | VOG | + | - | + | + | + | - | + | 3 | + | - | + | + | - | - | - | 2 |
| 4 | M | 56 | vertigo | 3 days | AR | - | - | - | - | - | - | - | 0 | - | - | - | - | - | - | - | 0 |
| 5 | M | 51 | vertigo | 5 days | AR | Unsuccessful AR Examination due to prior cataract surgery | | | | | | | | | | | | | | | |
| 6 | F | 59 | dizziness | 2 months | VOG | - | - | - | - | + | - | - | 0 | + | - | + | - | + | - | - | 0 |
| 7 | F | 58 | vertigo | minutes | AR | + | - | - | + | + | + | - | 0 | + | - | - | + | + | - | - | 1 |
| 8 | M | 46 | dizziness | 2 weeks | VOG | - | - | - | - | - | - | - | 4 | - | - | - | - | - | - | - | 2 |
| 9 | M | 54 | vertigo | 5 days | VOG | + | + | + | + | + | - | - | 0 | + | - | - | - | + | - | + | 0 |

*Note. This table presents demographic and clinical data of nine patients who underwent both augmented reality (AR)-based and video-oculography (VOG)-based nystagmus examinations. Columns include sex, age, symptom duration, test sequence (whether AR or VOG was performed first), and individual eye movement parameters assessed during each test. The presence (+) or absence (−) of abnormalities is documented for central vestibular pathology (Central), horizontal saccades (SacH), vertical saccades (SacV), horizontal smooth pursuit (PurH), vertical smooth pursuit (PurV), horizontal gaze fixation (GazH), and vertical gaze fixation (GazV). Patient discomfort was recorded using the Visual Analog Scale (VAS, 0–10). One patient had an unsuccessful AR examination, indicated in the table.*
