# Supplementary material for: Wearable Augmented Reality for Nystagmus Examination in Patients With Vertigo: Randomized Crossover Usability Study
Source: J Med Internet Res. 2025 Nov 11;27:e75327. doi: 10.2196/75327 (PMC12648123; doi:10.2196/75327)

**Multimedia Appendix 5. Heatmap of Agreement Rates Between AR and VOG Signals.** This heatmap illustrates the agreement rates (%) between augmented reality (AR)-based and video-oculography (VOG)-based eye movement signals for central vestibular pathology, saccades (horizontal [sacH], vertical [sacV]), smooth pursuit (horizontal [purH], vertical [purV]), and gaze fixation (horizontal [gazH], vertical [gazV]). Darker shades indicate higher agreement. Among 8 participants (56 data points), the highest agreement was for central vestibular pathology, purV, and gazH (87.5%), while sacV had the lowest (62.5%), highlighting variability in signal consistency.


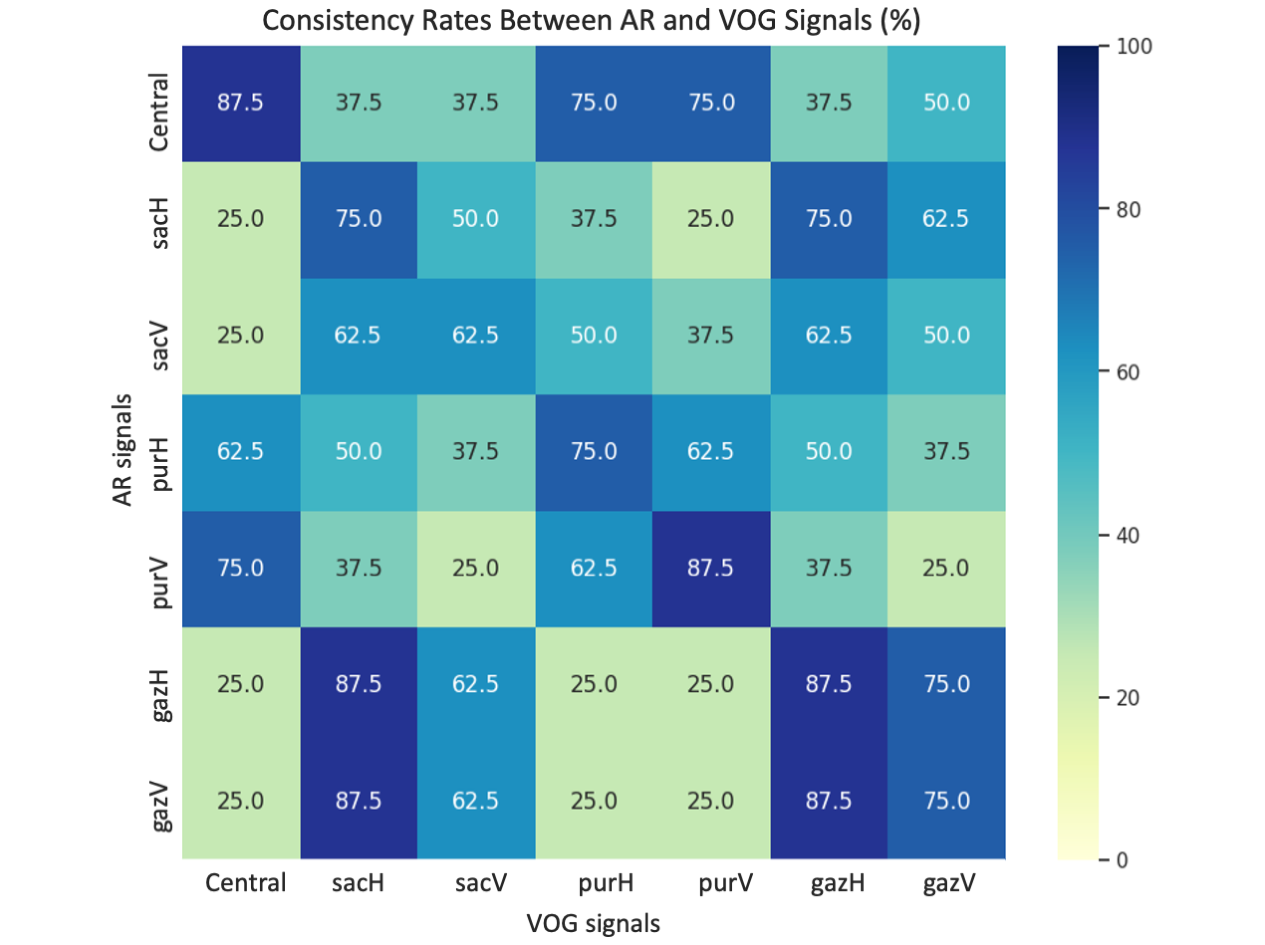

Supplement: Multimedia Appendix 5 [file jmir_v27i1e75327_app5.docx]
