# Supplementary material for: Wearable Augmented Reality for Nystagmus Examination in Patients With Vertigo: Randomized Crossover Usability Study
Source: J Med Internet Res. 2025 Nov 11;27:e75327. doi: 10.2196/75327 (PMC12648123; doi:10.2196/75327)
Supplement: Multimedia Appendix 6 [file jmir_v27i1e75327_app6.docx]

**Multimedia Appendix 6. Confusion matrix of AR-based vs VOG-based classifications of suspected central vestibular pathology (n=48 paired assessments)**

|  | AR Positive | AR Negative |
| --- | --- | --- |
| VOG Positive | 9 | 2 |
| VOG Negative | 9 | 28 |

*Note. This confusion matrix aggregates all 48 paired oculomotor assessments (six signal dimensions per participant). “AR Positive” and “AR Negative” denote the interpreter’s binary classification of suspected central pathology by the AR system; “VOG Positive” and “VOG Negative” correspond to the VOG benchmark. Cells indicate counts of true positives (TP = 9), false negatives (FN = 2), false positives (FP = 9), and true negatives (TN = 28).*
